# The Position That Dental and Oral Surgery Is Destined to Occupy in America

**Published:** 1881-04

**Authors:** Truman W. Brophy

**Affiliations:** Clinical Lecturer on Dental and Oral Surgery in Central Free Dispensary, Rush Medical College, Chicago


### Selections.

THE POSITION THAT DENTAL AND ORAL SURGERY IS DESTINED TO OCCUPY IN AMERICA. BY TRUMAN W. BROPHY, M.D., D.D.S., Clinical Lecturer on Dental and Oral Surgery in Central Free Dispensary, Rush Medical College, Chicago. (Read before the New York Odontological Society, February 15, 1881.)

The rapid advancement of the art and science of dental and oral surgery during the past quarter of a century has probably not been surpassed in the annals of history. The earnest, thoughtful, progressive practitioner, who loves his vocation and desires to aid in its advancement, cannot find another subject upon which his energies may be expended to such advantage as in laboring to elevate dentistry and place it before the whole world side by side with the other departments of art and science. What is dentistry, or —a better name,—dental and oral surgery, as recognized by the world to-day? (I say dental and oral surgery because the term more clearly represents, or indicates, the tissues upon which the educated dental surgeon is called on to operate.) By a few of the more intelligent it is, as it should be, accepted as a science as well as an art; but by the greater number of well-educated people, —people possessing extensive information on nearly all other subjects,—cultured people, some, indeed, who occupy prominent places in the learned professions,—men distinguished for their classical education and attainments,—really believe that the practitioner of dentistry is pursuing a menial vocation, and is simply an ordinary mechanic. Such being the views of some of the more intellectual, what may we expect from those who occupy less prominent places in society?

Let us look for a moment at the condition of dentistry in this country and see if our friends are not, to some extent, at least, justified in their opinion of us. There are, in thirteen States, laws to regulate the practice of dentistry; in the other States, including Illinois, any one who so elects, even though he can neither read nor write, may place out a sign and enter upon its practice. So long as this condition of things is permitted to exist it is not reasonable to expect the laity to recognize dentistry as having any claim to the name of a profession or any part thereof, or to place it on a par with the universally-accepted learned professions of the world.

The work so well and faithfully performed by our eminent American dentists in conducting institutions of dental learning, as well as in establishing and successfully maintaining dental associations, has resulted in Americans justly receiving acknowledgments from the whole dental world as excelling in this department of art and science. Upon these men who have conscientiously labored, devoting their lives and fortunes to their efforts to advance a branch of science,—some of whom, owing to their assiduous exertions, now rest in untimely graves,—too much honor cannot be bestowed.

Notwithstanding the great advancement made in dentistry by the efforts of the energetic men who are and have been engaged in its practice, I cannot but think that the course so long pursued in regard to dental education and association falls far short of what it should be. There is no doubt that dental surgery is a specialty of medicine; and holding that belief, we should at once take steps to place our specialty side by side with other special branches of medical learning. Can this be accomplished? What shall we do to verify the clause which appears in the Code of Ethics of the American Dental Association, to wit, "Dental surgery is a specialty in medical science," etc., etc.? Can we secure for dental and oral surgery recognition as a medical specialty equal to that so universally accorded to ophthalmology, otology, gynæcology, etc.? Most certainly we can! Can it be done by establishing new societies of dental surgeons and admitting only men who are M. D.'s? Not by any means. While all credit should be given to the distinguished gentlemen of Boston,—doctors of medicine practicing dental and oral surgery,—who have wisely formed "The Society for the Advancement of Oral Science," its good results will probably be largely of a local nature, though its influence may extend far beyond the city of Boston and become a stimulus to similar societies. It will be impossible to place the specialty of dental and oral surgery on a par with other medical specialties by organizing such societies, it matters not how eminent their members may be, how high their standard, or how exacting their requirements for admission to their ranks.

There is, undoubtedly, but one way by which dental and oral surgery can be placed in the position of equality with the other recognized medical specialties, and that is bringing it within the province of the American Medical Association, made up as it is of the most distinguished medical men upon the American continent; men, indeed, representing every State in this broad Union; men representing every specialty of the science of medicine except one. Who are these medical specialists that are so negligent as to suffer their specialty to be the only one without representation in this the highest medical association in their land? Who are these medical men who have not considered their specialty of sufficient importance to carry their banner to the front, and place it in line with those of other specialties? None others than the doctors of dental and oral surgery. Is this not true? And yet we claim to be medical specialists. I believe the time has come when we should cast off the scales from our eyes, realize our position as medical men, and immediately take steps to place our specialty where it belongs—in the American Medical Association. Warm friends will greet us there,—men whose names are familiar to every medical practitioner and student in every civilized nation of the earth. Such action will no doubt be taken in May next, at Richmond, Va., where the American Medical Association holds its next annual meeting; and when the section of dental and oral surgery is established, it will be the forerunner of a new era, in which dentistry in America will be elevated to a higher plane than it has heretofore attained. There will probably be opposition on the part of some of the members of the Medical Association to this procedure, but I am firm in the belief that this highly intelligent body will recognize the importance of such a section, and, therefore, receive us most cordially. We shall probably meet with opposition from our dental friends, who will argue that we should stand by dentistry for what it is. They will also speak of the absurdity of placing dentistry where physicians and surgeons may, if they desire, enter upon its practice, even though they are ignorant of all the manipulations so essential to a competent dental operator. To such arguments—and as many more as our friends deem advisable to enter into—we shall answer at the next meeting of the American Dental Association, which will occur in New York City the coming August.

Placing dental and oral surgery in a position where the whole world must accept it as a department of medicine, will make it a more exalted vocation, and a greater number of men of higher intellectuality will be found in our ranks than would ever come under the present *régime*. It will add a new impetus to thought and investigation. It will be an incentive to young men who aim to be thoroughly qualified to practice dentistry, to educate themselves as doctors of medicine in conjunction with their dental training. With this accomplished we shall be accepted as medical men practicing a specialty of medicine. We shall no longer be erroneously called the "dental profession," any more than ophthalmologists should be said to belong to the ophthalmological profession or dermatologists to the dermatological profession; but we shall, in common with the others, be medical men,—doctors of medicine with all the title implies,—practicing a specialty thereof not second in importance to any.

The plan here proposed, and which will in all probability be carried out, is as follows: All regular physicians practicing dental and oral surgery are requested, if they are in sympathy with this movement, to join their county medical society (if they are not already members), get their credentials to the American Medical Association, to go Richmond, Va., where it convenes in May next, when a section on dental and oral surgery will be organized, and application be made to the Association for admission. If there be any prominent member opposed to this course, I have neither seen him nor heard from him. I have in my possession letters, in answer to mine, from some of the most distinguished members, requesting their views of the scheme, containing such expressions as follows: "I wonder why this was not done long ago." "Dental surgery is as much a department of medicine as ophthalmology, and consequently should have representatives in the American Medical Association, *provided they are medical men.*" "I am glad to see that you have at last realized what you are, and are now ready to do your duty, as you have expressed it to me, and return home to your mother who gave you birth."

I might quote pages from eminent men engaged in general practice, as well as from those engaged in all the specialties, including ours, but these I have given are fair samples of the whole number. That I may not be misunderstood, I wish to say that the American Association is, and probably always will be, the only truly national association of dentists in this country, and it is indeed a high honor to any dentist to hold membership therein. The time will doubtless come when every member of this association will be a medical man,—an M.D. An impression must not go out to the effect that we are in the least dissatisfied with the American Dental Association. Not so by any means. The grand work achieved by that body will adorn the pages of the history of dentistry many years after our children's children have passed away, and other minds are grappling with and endeavoring to solve the great and ever-absorbing problem of professional advancement.

While a large majority of its members are not medical men, we shall feel that it holds a position corresponding to that occupied by the American Gynæcological Association, the American Dermatological Association, etc.; but, like these latter associations, we, too, should have members of our specialty in the American Medical Association, the *only* great national association for all medical practitioners, either general or special.

Injustice would be done to ourselves if this paper were concluded without claiming that we shall impart much information to our associates in other departments of medical science, and furnish them with valuable papers upon the *most prevalent* of *all* diseases,—diseases which lead to more suffering than any others known to civilized man,—diseases, indeed, the etiology of which the general practitioner has studied but little, for want of time, but which not infrequently originating in distant organs of the body, manifest themselves in the teeth or maxilla, and *vice versa*.

The facial neuralgias which prevail to such an extent are largely due to dental lesions, and too often irretrievable losses have been sustained by patients without just cause.

It is for these, with other reasons previously stated herein, that a section should be formed on dental and oral surgery in the American Medical Association. No one can justly claim that the American Medical Association conflicts in any way with the good work accomplished by the American Dermatological Association, or the American Ophthalmological Association, or other associations whose work is upon special subjects pertaining to the science of medicine.

Such will be the relation of the American Medical Association to the American Dental Association. Working together hand in hand in one common cause, with one motive, we shall go forward, mutually acquiring that knowledge which will make us better, and enable us to stand before the world conscious of having used our combined talents to the extent of our ability to advance the most noble of all professions,—whose existence is dedicated to the alleviation of human suffering and the promotion of the happiness of man.—*Dental Cosmos*, March, 1881.

